# Post-Transcriptional Silencing of Flavonol Synthase mRNA in Tobacco Leads to Fruits with Arrested Seed Set

**DOI:** 10.1371/journal.pone.0028315

**Published:** 2011-12-01

**Authors:** Monika Mahajan, Paramvir Singh Ahuja, Sudesh Kumar Yadav

**Affiliations:** Plant Metabolic Engineering Laboratory, Biotechnology Division, CSIR-Institute of Himalayan Bioresource Technology, Council of Scientific and Industrial Research, Palampur, India; United States Department of Agriculture, United States of America

## Abstract

Flavonoids are synthesized by phenylpropanoid pathway. They are known to participate in large number of physiological and biochemical processes in plants. Parthenocarpy and male sterility has earlier been reported by silencing chalcone synthase (CHS) encoding gene. Silencing of *CHS* has blocked the synthesis of most of useful flavonoids including flavan-3-ols and flavonols. Also, these studies could not identify whether parthenocarpy/male sterility were due to lack of flavan-3-ols or flavonols or both. Flavonol synthase (FLS) is an important enzyme of flavonoid pathway that catalyzes the formation of flavonols. In this article, we propose a novel strategy towards the generation of seedless or less-seeded fruits by downregulation of flavonol biosynthesis in tobacco (*Nicotiana tabacum* cv Xanthi) through post-transcriptional gene silencing (PTGS) of FLS encoding mRNA. The *FLS* silenced lines were observed for 20-80% reduction in FLS encoding gene expression and 25–93% reduction in flavonol (quercetin) content. Interestingly, these *FLS* silenced tobacco lines also showed reduction in their anthocyanidins content. While the content of flavan-3-ols (catechin, epi-catechin and epi-gallocatechin) was found to be increased in *FLS* silenced lines. The delayed flowering in *FLS* silenced lines could be due to decrease in level of indole acetic acid (IAA) at apical region of their shoots. Furthermore, the pollen germination was hampered and pollens were unable to produce functional pollen tube in *FLS* silenced tobacco lines. Pods of *FLS* silenced lines contained significantly less number of seeds. The *in vitro* and *in vivo* studies where 1 µM quercetin was supplied to germination media, documented the restoration of normal pollen germination and pollen tube growth. This finding identified the role of flavonols particularly quercetin in pollen germination as well as in the regulation of plant fertility. Results also suggest a novel approach towards generation of seedless/less-seeded fruits via PTGS of FLS encoding gene in plants.

## Introduction

Flavonoids are plant secondary metabolites and are widespread throughout the plant kingdom. This category of compounds includes both pigments such as chalcones and anthocyanins as well as colorless molecules such as flavanones, flavones, and flavonols.

Presence of flavonoids has also been documented in pollen and pistils of many plant species [1]. Amongst flavonoids, flavonols particularly kaempferol has been found essential for pollen germination and tube growth in petunia (*Petunia hybrida*) and maize [2], [3]. Hence, flavonols are considered to play an important role in fertility and sexual reproduction in plants. Transgenic plants suppressed for gene encoding chalcone synthase (CHS), the first enzyme in the flavonoid pathway were instrumental in identifying such an essential role of flavonols in pollen function. The lack of CHS protein in both petunia and maize anthers has resulted in white pollen. These pollens were devoid of flavonols and were unable to germinate or produce a functional pollen tube on self-pollinations [2], [4]. Further evidence for a role of flavonols in sexual reproduction was provided by the male sterile petunia white anther (*wha*) mutant. This mutant showed complementation by an introduction of a functional CHS cDNA [5]. The reduced level of flavonoids in tomato led to lack of functional fertilization and resulted into seedless fruits [6]. Production of such fruits can be a desirable trait for several important crop plants that are of great value for consumers as well as for the processing industry.

**Figure 1:**
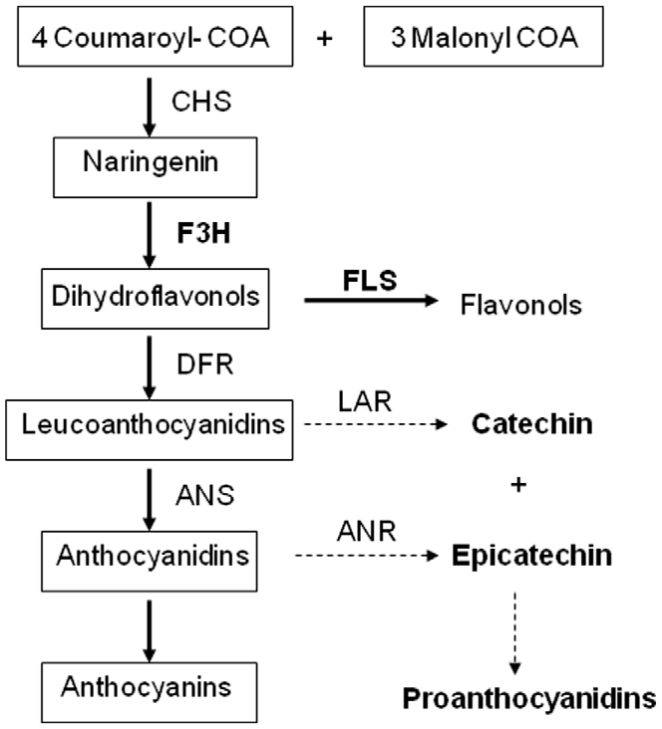
Schematic overview of the flavonoid biosynthesis pathway in plants. The pathway normally active in tobacco leaves and inflorescence, leading to flavonols and anthocyanin production, is indicated by solid arrows. Whereas dashed arrows indicate the minor pathway in tobacco that leads to flavan-3-ols (catechin and epicatechin) synthesis. Abbreviations: CHS, Chalcone synthase; CHI, chalcone isomerase; F3H, flavanone 3-hydroxylase; FLS, flavonol synthase; DFR, dihydroflavonol 4-reductase; ANS, anthocyanidin synthase; LAR, leucoanthocyanidin reductase; ANR, anthocyanidin reductase.

Tobacco (*Nicotiana tabacum*) belongs to Solanaceae family is an important model plant and is being used for the genetic modification of flavonoid pathway. Flavonoids are synthesized through the phenylpropanoid pathway [7]. The precursors for synthesis of most flavonoids are malonyl-CoA and p-coumaroyl-CoA. This pathway is initiated by an enzymatic step catalyzed by chalcone synthase (CHS) resulting in chalcone production, which is further converted by chalcone isomerase to naringenin. Further downstream in the main pathway, there is a competition between enzymes flavonol synthase (FLS) and dihydroflavonol 4-reductase (DFR) for the common substrate dihydroflavanols. The FLS and DFR catalyzed reactions lead to the production of flavonols and anthocyanidins/catechins, respectively (Fig. 1). An increased level of anthocyanins in flowers of transgenic antisense *FLS* of petunia and tobacco has been reported [8], [9], [10]. This temporal regulation of enzyme activity that is using the same substrate is an attractive way to prevent substrate competition for dihydroflavanols to be used either for anthocyanin, catechin or flavonol biosynthesis.

The possible role of flavonoids in free indole acetic acid (IAA) distribution has also been proposed. Aerial parts of a plant especially the young developing leaves are an important source of free IAA for rest of the plant [11]. Free IAA is known to enter into roots from shoots and transported through central tissue of root towards the tip. Quercetin, kaempferol, and bestatin have been identified as the most active flavonoids acting as regulators for the transport of endogenous free IAA, and thereby affecting root development in plants [12], [13], [14]. These later flavonoids have also been screened for their ability to block the binding of a synthetic auxin transport inhibitor, naphthylphthalamic acid (NPA) and to inhibit auxin transport from hypocotyls segments [11], [15].

Earlier studies have documented the parthenocarpy in tomato and male sterility in petunia by silencing chalcone synthase (CHS) encoding gene [6], [16]. Silencing of CHS had blocked the synthesis of most of flavonoids and that is undesired character as flavonoids are very important antioxidants. To identify whether blocking the whole pathway is important to obtain parthenocarpy/male sterility as was done in tomato and petunia through silencing *CHS* or only reduction in flavonols by silencing *FLS* would be sufficient to obtain parthenocarpy/less-seeded fruits. In this study, we have silenced *FLS* to reduce flavonols (quercetin) content in tobacco (*Nicotiana tabacum* cv Xanthi). These *FLS* silenced tobacco were used to analyze the effect on flavonoid biosynthesis. Silenced lines were further used to examine the role of flavonols (quercetin) in plant reproduction and fruit development. Silencing of *FLS* leads to the development of fruits with arrested seed set. Hence, a novel approach of obtaining fruits with significantly less number of seeds through *FLS* silencing that reduced quercetin content has been documented.

## Results

### Downregulation of *FLS* gene expression in tobacco through RNAi approach

**Figure 2:**
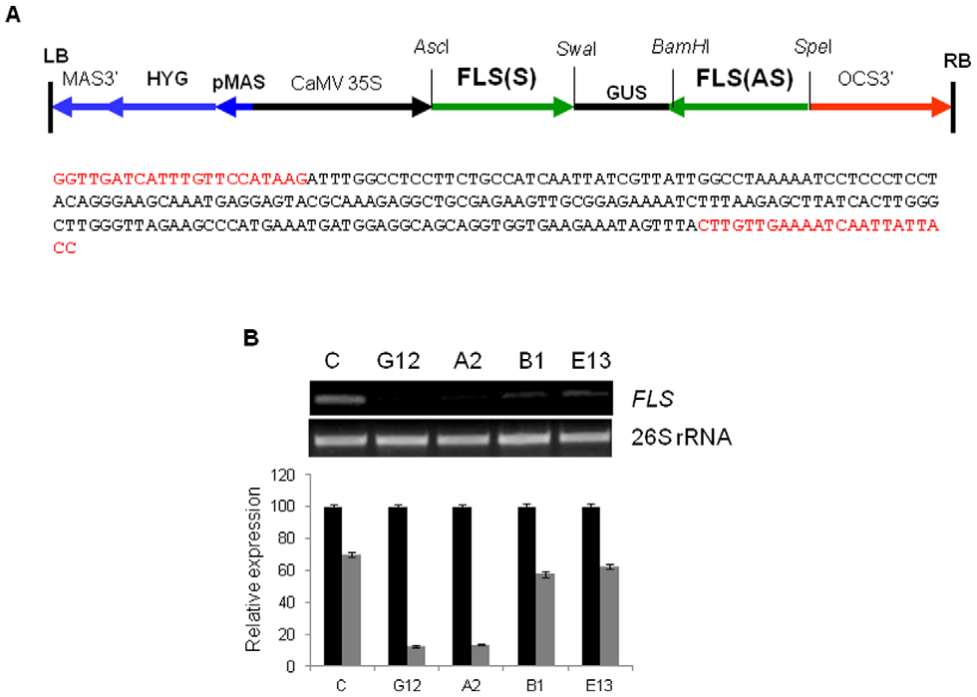
Generating FLS silenced tobacco lines and their confirmation. A, Schematic drawing of the *FLS* hairpin (hpRNAi) construct. Transgene expression was under the control of a CaMV 35S promoter. An inverted repeat was generated by cloning a sense *FLS* cDNA fragment (233 bp) followed by the similar cDNA sequence encoding tobacco *FLS* in anti-sense orientation in pFGC1008 vector backbone. The conserved sequence of 233 bp given below was used in developing hpRNA binary vector (pFGC1008) for RNA interference study in tobacco. B, Semiquantitative RT-PCR analysis. Steady-state mRNA levels of tobacco *FLS* relative to the housekeeping gene 26S rRNA were measured in leaf tissue of silenced transgenic (G12, A2, B1, E13) and control lines. Below gel picture, bar diagram shows relative transcript levels of the respective amplified bands. Expression analysis was repeated at least three times and representative one time gel pictures are presented. Data are means of three measurements ± SD. Black and grey bars show 26S rRNA and flavonol synthase (FLS) enzyme transcript levels, respectively. The first two silenced transgenic lines G12 and A2 showed about 80% reduction in FLS expression while other two silenced lines B1 and E13 showed up to 18-20% reduction in FLS expression. Values represent the average of three biological replicates, each with three technical replicates. C, control; G12, A2, B1, E13, different silenced transgenic lines.

To downregulate flavonol synthesis in tobacco and to see the influence of such silencing on plant function, *FLS* hpRNAi gene construct was prepared (Fig. 2A). EST database searches and southern hybridization signals have suggested the presence of two flavonol synthase (*FLS*) gene family members (FLS 1; DQ435530.1 and FLS; AB289451.1) in tobacco. Both sequences were aligned using CLUSTAL W (Fig. S1). The 233 bp conserved region of both sequences was used in developing hpRNA binary vector for RNA interference study in tobacco (Fig. 2A). To create an inverted repeat construct, this *NtFLS* cDNA fragment was cloned in sense and antisense orientation on either side of GUS intron in pFGC1008 vector (Fig. S2). The resulting RNAi construct (pFGC-FLS) was introduced in tobacco (*Nicotiana tabacum* cv Xanthi) using *Agrobacterium*-mediated leaf disc transformation. This *FLS* RNAi construct was expressed under the control of a constitutively enhanced cauliflower mosaic virus (CaMV) 35S promoter, and, therefore, it was expected that the transgene effect would influence the flavonoid pathway in all parts of the tobacco plant.

The transformants were firstly confirmed for PCR-positive *FLS* RNAi cassette using vector specific primers (Fig. S3). The positive transgenic plants were further used for flavonol synthase (FLS) encoding gene expression analysis. The *FLS* transcript expression in control and silenced transgenic lines was studied through reverse transcriptase-PCR. The constitutively expressed 26S rRNA was used as an internal standard in expression analysis. Out of 10 PCR-positive transgenic lines, only 4 lines showed downregulation in transcript expression of *FLS* gene. The two *FLS* silenced lines G12 and A2 showed up to 80% decrease in expression levels of *FLS* gene as compared to control. In contrast, a relatively small decrease in *FLS* expression of about 20-22% was found in other two *FLS* transgenic lines B1 and E13 (Fig. 2B). The decreased flavonol synthase gene expression was found to segregate with the *FLS* RNAi cassette in lines G12, A2, B1 and E13 tested in their successive T1 and T2 generation. Based on hygromycin resistance, homozygous transgenic lines were selected for further analysis.

### Phenotypic characterization of FLS silenced tobacco

**Figure 3:**
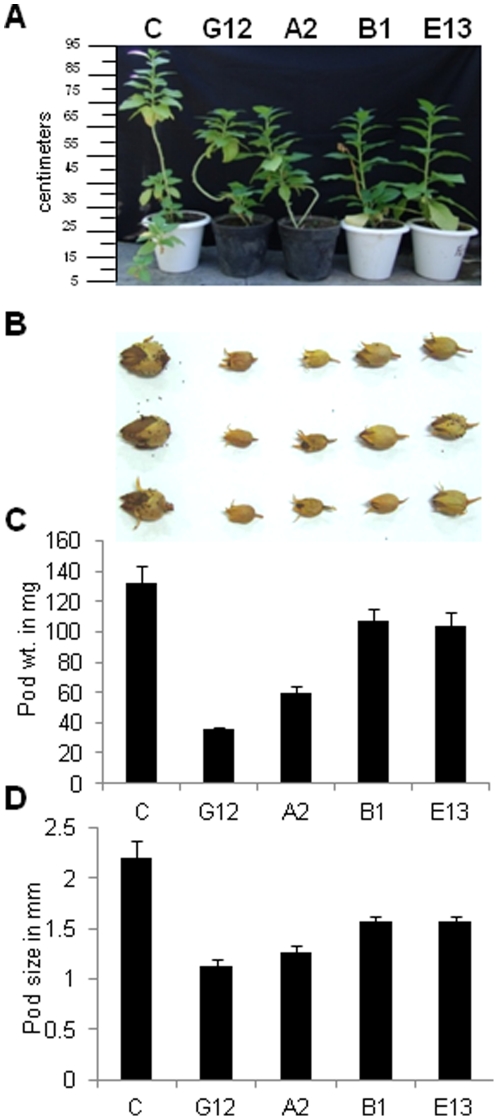
Morphological characterization and yield parameters of *FLS* silenced tobacco lines compared to control. A, *FLS* silenced transgenics lines G12, A1, B1, and E13 were smaller in height as compared to control tobacco plant (*Nicotiana tabacum* cv xanthi). Flowering was delayed in FLS silenced transgenics. Scale bar in centimeter is shown on left side of the image. B, Pods derived from control flowers that were self pollinated grew to normal size. Whereas, self pollinated silenced transgenic lines G12, A2, B1and E13 yielded smaller fruits. In *FLS* silenced lines pods and seed development was arrested, whereas control (C) tobacco pods had a normal seed set. C, Pod weight in milligrams and D, pod size at equatorial cross section in millimetres of control (C) tobacco plant and of *FLS* silenced transgenic tobacco lines (G12, A2, B1 and E13). Both pod weight and pod size was reduced in all silenced transgenic lines as compared to control. Values represent mean values ± SD (n = 5).

The *FLS* silenced tobacco lines were smaller in height to that of control plant (Fig. 3A). Further, *FLS* silenced lines showed a delayed fruit development and yielded smaller fruits (Fig. 3A and 3B). The small fruits (pods) of *FLS* silenced lines A2, G12, B1 and E13 contained very less number of seeds as compared to control tobacco (Table 1). This has suggested the arrest in seed set due to *FLS* silencing in tobacco. The pods of transgenic lines A2 and G12 have produced significantly very less number of seeds. The average number of seeds per pod was 143, 300, 1010 and 1160 in G12, A2, B1 and E13 silenced lines as compared to 1417 seeds per pod of control tobacco plant. Additionally, average number of pods per plant was also very less in case of *FLS* silenced transgenic lines compared to control plant. Pods per plant were found to be 4, 5, 8 and 9 for G12, A2, B1 and E13 lines respectively as compared to 11 pods for control tobacco (Table 1). Further, the pod size as well as pod weight was also reduced significantly in all four transgenic lines as compared to control. The silenced transgenic lines A2 and G12 showed higher decrease in pod weight and pod size as compared to pods of B1 and E13 transgenic lines and control tobacco plant. Pod weight was 35.33 mg, 59.67 mg, 107.67 mg and 110 mg for G12, A2, B1 and E13 *FLS* silenced lines respectively compared to 132 mg pod weight of control tobacco (Fig. 3C). Similarly, the pod size of G12, A2, B1 and E13 silenced lines was found to be 1.13 mm, 1.27 mm, 1.57 mm and 1.6 mm respectively as compared to 2.2 mm of control tobacco pod (Fig. 3D).

**Table 1:** Comparative fruit characteristics of control and FLS silenced tobacco.

	control	G12	A2	B1	E13
Seeds per fruit (pod)	1417±104	143±40	300±50	1010±36	1160±53
Pods (n)	11	4	5	8	9

Seeds per fruit (pod) and number of pods per plant were determined in control and FLS silenced transgenic lines G12, A2, B1 and E13. Data is the mean of three replications ± SD.

### Flavonoid biosynthetic pathway genes expression and flavonoid levels in FLS silenced lines vis-à-vis control plant

The effect of *FLS* silencing on transcript level of other flavonoid biosynthetic pathway genes was analyzed through RT-PCR. Although FLS encoding gene expression was reduced in shoot and root tissues of silenced transgenic lines, but no significant difference in expression levels of other flavonoid biosynthetic pathway genes encoding chalcone synthase (CHS), chalcone isomerise (CHI), flavonol-3-hydroxylase (F3H), & anthocyanin synthase (ANS) was observed in shoot and root tissues of *FLS* silenced transgenic lines compared with control (Fig. S4).

**Figure 4:**
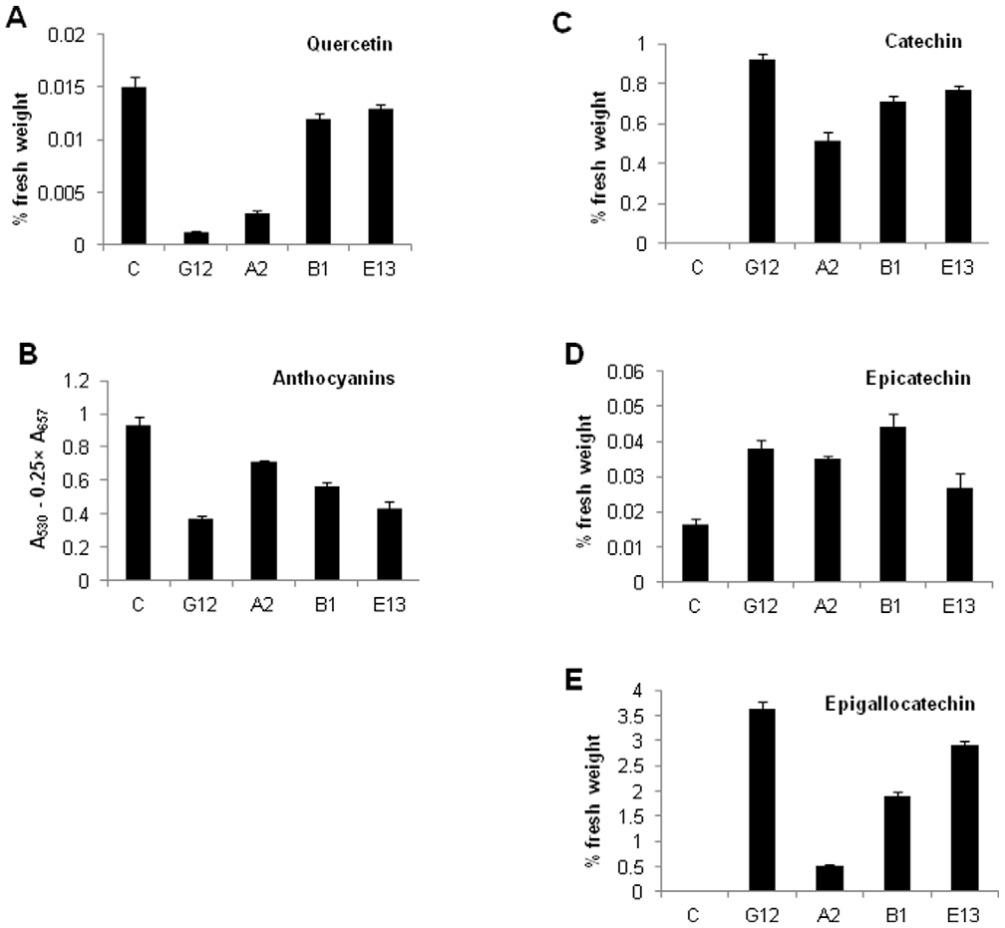
Comparison of flavonoid levels between control and *FLS* silenced transgenic tobacco. A, Flavonol content in leaf extract of control (C) and different *FLS* silenced transgenic tobacco lines (G12, A2, B1 and E13). More than 80% reduction in flavonol content was observed in silenced transgenic lines (A2 and B12) through HPLC analysis. Other two lines (B1 and E13) showed about 20% reduction in flavonol content. B, Anthocyanin content in methanolic extracts of flowers of control (C) and different silenced transgenic lines. A_530_, absorption at 530 nm; A_657_, absorption at 657 nm. C, Catechin; D, Epicatechin; E, Epigallocatechin content determined through HPLC analysis in leaf extract of different *FLS* silenced transgenic lines relative to the control (C). All three contents i.e. catechin, epicatechin & epigallocatechin were found to increase to various degrees in silenced transgenic lines as compared to control (C) tobacco plant. Error bars represent the SD of the average from a total of three measurements using two independent biological replicates.

As FLS enzyme competes with dihydroflavonol 4-reductase (DFR) for the common substrate dihydroflavonols, its downregulation might be affecting the levels of other flavonoids. Four lines G12, A2, B1 and E13 with reduced level of *FLS* gene expression were chosen for estimation of flavonoid contents. Since FLS enzyme leads to the formation of flavonols, quercetin content was also measured in leaves of silenced and control plants. Based on HPLC analyses of leaf extract, the decrease in quercetin content was observed in these silenced tansgenic lines as compared to control tobacco plant. For all transgenic lines, the observed decrease in quercetin content was well correlated with expression data. The G12, A2, B2 and E13 lines showed 93%, 80%, 27% and 25% reduction in their quercetin content respectively as compared to control tobacco plant (Fig. 4A). Therefore, A2 and G12 were regarded as lines with “strong” phenotype whereas B2 and E13 as “weak” phenotype.

To see the effect of flavonols particularly quercetin content reduction on the flux of flavonoid towards anthocyanidin or flavan-3-ols (catechin, epicatechin and epi-gallocatechin) formation, these contents were also measured in silenced transgenic lines vis-à-vis control plants. Anthocyanin content was decreased by 58%, 22%, 29% & 47% in G12, A2, B1 and E13 silenced lines respectively as compared to control tobacco plant (Fig. 4B). Among the silenced transgenic lines, the anthocyanin content was higher in A2, followed by B1, E13 and G12. Interestingly, HPLC analysis showed an increase in catechin, epi-catechin and epi-gallocatechin contents of silenced transgenic lines compared to control plant. The silenced transgenic lines G12, A2, B1 and E13 showed 98%, 45%, 65% & 74% increase in catechin content respectively as compared to control plant. Catechin content was very low in control plants (Fig. 4C). Similarly, epicatechin content was increased by 111%, 94%, 128% & 38% in G12, A2, B1 and E13 silenced lines respectively (Fig. 4D) and epigallocatechin content was increased by 351%, 49%, 157% & 258% in G12, A2, B1 and E13 silenced lines respectively (Fig. 4E) as compared to control plant.

### Pollen tube growth and fertility in FLS silenced transgenics

**Figure 5:**
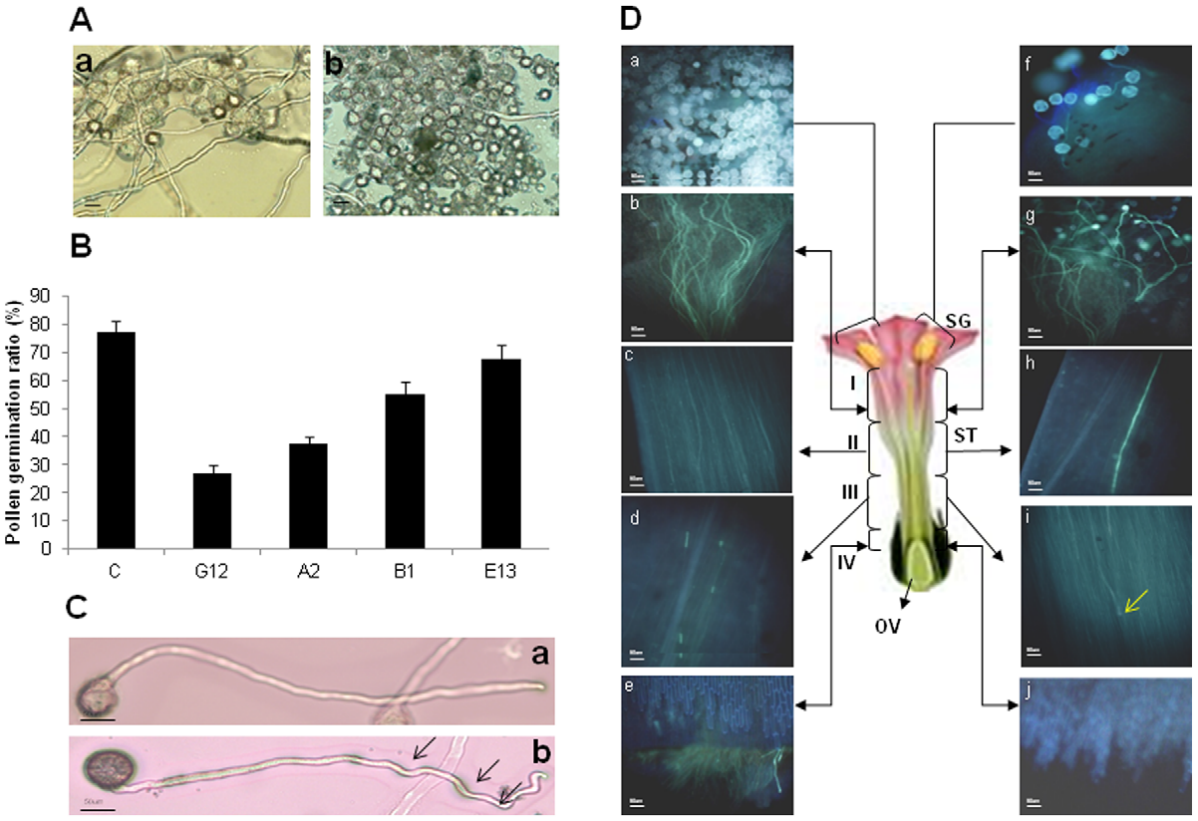
*In vitro* and *in vivo* pollen germination assays of control and FLS silenced transgenic tobacco. A, *In vitro* pollen germination assay of pollens from control tobacco plant (a) and from *FLS* silenced transgenic line G12 (b). These pollens were taken from freshly dehiscent anthers and were dapped on slides containing pollen germination medium (GM). Pictures were taken after 4 h incubation. The pollen germination rate was reduced in silenced transgenic line as compared to control. B, Graph depict the germination frequency (pollen germination percentage) of pollens from control tobacco plant and from *FLS* silenced transgenic tobacco lines (G12, A2, B1 and E13) on GM after 4 h of incubation. Pollen germination frequency was found to be reduced in silenced transgenic lines as compared to control tobacco. Only tubes longer than half the size of pollen grains was judged as germinated. Values are mean of three replications where tube length of 50 to 100 pollen grains was measured and are represented as mean ± SD. C, Shape and surface characteristics of pollen tubes. Ten pollen tubes of each transgenic and control plants were analyzed. The foremost part of a pollen tube of control tobacco plant show a smooth, straight shape (a), whereas more that 80% (out of 10, 8-9 pollen tubes) of germinated pollen tubes of *FLS* silenced transgenic tobacco show the kinked, and coiled shape (b) after 4 h of incubation on germination media. The arrows indicate the rough surface of the pollen tube. D, Histochemical staining of pollen tube growth in carpels after 2 days of pollination from control and *FLS* silenced line G12. Fertilized carpels were stained with aniline blue to specifically stain callose present in growing pollen tubes. Staining was conducted in control tobacco carpels after crossing with control plant pollens (a–e) and G12 line carpels after self-crossings (f–j). Callose in the pollen tubes is visible at the stigma (a and f). b, c, g and h, showing proliferation of pollen tube growth in the middle of the style. Pollen tubes of G12 *FLS* silenced line grew only nine-tenths of the way down the style (i). The tips of the pollen tubes are swollen in G12 *FLS* silenced line (shown by arrow). Pollen tubes are not visible at the base of the style in G12 *FLS* silenced line (j) as compared to control carpels (e). All micrographs are of the same magnification and the scale bar in A, C, and D pictures represent 50 µm. The pictures were taken under UV filter of florescent microscope equipped with Nikon digital camera Dxm 1200 C.

Here, we investigated through *in vitro* and *in vivo* experiments whether pollen germination and pollen tube growth were affected in *FLS* silenced transgenic tobacco lines. For *in vitro* experiment, pollens from control as well as *FLS* silenced transgenic lines were germinated on pollen germination media. After 4 h of incubation, significant reduction in pollen germination percentage of all *FLS* transgenic lines was observed compared to control. The representative picture of pollens of *FLS* silenced transgenic line G12 and control is shown (Fig. 5A). The pollen germination percentage was 26% (reduced by 74%), 37% (reduced by 63%), 73% (reduced by 27%) and 80% (reduced by 20%) in G12, A2, B1 and E13 silenced transgenic lines respectively as compared to control (Fig. 5B). In addition, more than 80% of the germinated pollen tubes of *FLS* silenced transgenic lines had a relatively rough surface and showed kinked and coiled shape compared to the control plant (Fig. 5C).

For *in vivo* pollen germination studies, fertilized carpels of control and *FLS* silenced G12 transgenic line were histochemically stained specifically for callose present in growing pollen tubes after 2 days of pollination. In control self-pollinated plants, pollen tubes were reached to the base of style after 2 days of pollination (Fig. 5D, a–e). Whereas, pollen tubes of *FLS* silenced G12 self-pollinated flowers did not grow well and did not reach to the base of style after 2 days of pollination. The *FLS* silenced line showed clear staining of callose in the stigma and absent further down, indicating the arrest in pollen tube germination. In *FLS* silenced line, the pollen tubes grew only to about nine-tenths of the way down the style, and tube tips were found somewhat swollen (Fig. 5D, f–j).

### Rescuing effect on pollen germination and fertility of FLS silenced lines upon quercetin supplements

**Figure 6:**
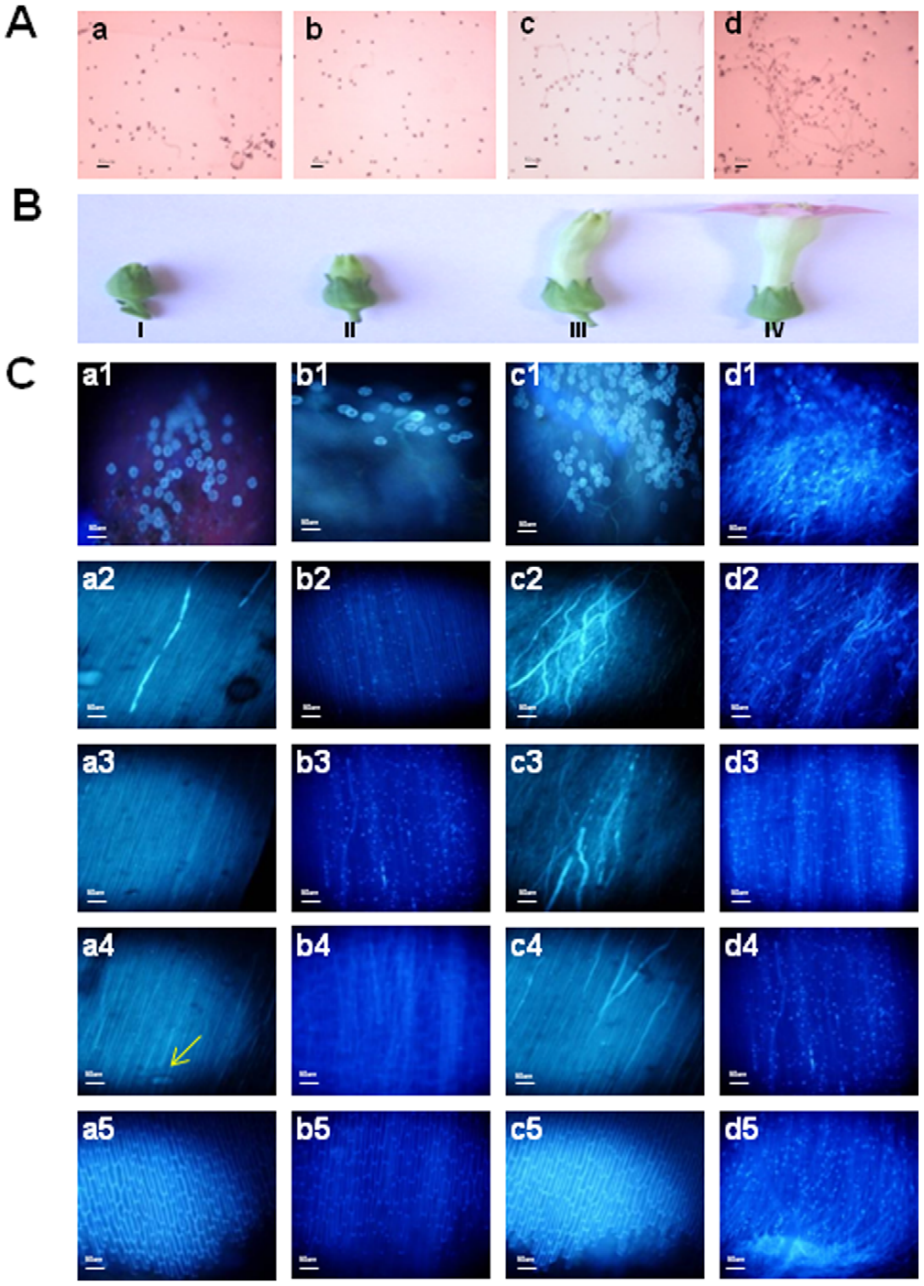
*In vitro* and *in vivo* pollen germination rescue assays of FLS silenced transgenic tobacco. A, Effect of quercetin on *in vitro* pollen germination rate of silenced transgenic pollens. Pollens were collected from freshly dehiscent anthers of *FLS* silenced tobacco plant and suspended in GM containing (a) only DMSO added to final concentration of 1 µM; (b) 10 nM quercetin; (c) 20 nM quercetin and (d) 1 µM quercetin. The pollen germination rate and pollen tube length was increased in GM containing 1 µM quercetin as compared to pollens that were exposed to 10 nM, 20 nM and with no quercetin. B, Different developmental stages of flower bud (stage I, II, III and IV) in tobacco (*Nicotiana tabacum* cv xanthi). It has been proven experimentally that auxin is mainly accumulated in anthers and stigma during stage II (the stage after bud formation when flower has not yet opened) of floral development [39]. C, Histochemical staining of pollen tubes growth in carpels of floral buds (stage II) from silenced transgenic lines exposed to different concentrations of quercetin i.e. 10 nM, 20 nM and 1 µM through pollen maturation media (PM). After 2 days of pollination, carpels were stained with aniline blue to specifically stain callose present in growing pollen tubes. Pollen tube growth in self pollinated carpels of floral bud of silenced transgenic lines exposed only to PM without any treatment (a1-a5); carpels of floral bud exposed to PM containing 10 nM quercetin (b1-b5); 20 nM (c1-c5) and 1 µM (d1-d6) quercetin. Pollen germination is visible at stigma region (a1, b1, c1 and d1). It was found to be maximum with 1 µM (d1) and least in silenced pollens with no treatment (a1). The pictures a2-a4, b2-b4, c2-c4 and d2-d4 show proliferation of pollen tube growth in the middle of the style. The pollen tubes grew only to nine-tenths of way down the style after 2 days of pollination in case of silenced flora buds exposed to 10 nM (b2-b4), 20 nM (c2-c4) quercetin and with no treatment (a2-a4). Whereas pollen tubes are reaching the base of the style in case of floral buds exposed to 1 µM quercetin (d2-d5). On the other hand, in untreated buds and in buds exposed to 10 nM, 20 nM quercetin, no pollen tubes are seen in carpels reaching the base of style (a5, b5 and c5) within the same time period. All micrographs are of the same magnification and the scale bar in A, and C pictures represent 50 µm. The pictures were taken under UV filter of florescent microscope equipped with Nikon digital camera Dxm 1200.

To confirm that the arrest in pollen germination of *FLS* silenced lines was due to lower levels of quercetin, *in vitro* and *in vivo* experiments were performed with *FLS* silenced transgenic pollens by supplying quercetin through media. For *in vitro* experiments, pollens from *FLS* silenced transgenic flowers were germinated on pollen germination media that contained various concentrations of flavonol (quercetin) as 10 nM, 20 nM and 1 µM. Pollen germination rate as well as pollen tube length was found to be increased in 1 µM of quercetin supplemented medium (Fig. 6A, d) as compared to 10 nM (Fig. 6A, b), 20 nM (Fig. 6A, c) and without any quercetin (Fig. 6A, a) supplemented media. With 20 nM quercetin, only some of the pollens showed germination.

*In vivo* pollen germination assay was also performed, where tobacco flower bud at stage II (Fig. 6B) was exposed to pollen maturation (PM) media that contained various concentrations of flavonol (quercetin) as 10 nM, 20 nM and 1 µM. For control, buds were exposed to PM medium alone with no supplements of quercetin. Upon maturation in the media, flower buds were emasculated and pollinated. After two days of pollination, pistils from all the flowers were harvested and histochemically stained for callose staining of pollen tubes. Pollen tube growth was very efficient in flower buds that were exposed to 1 µM quercetin (Fig. 6C, d1-d5) as compared to flower buds allowed to mature in media with 10 nM (Fig. 6C, b1-b5), 20 nM (Fig. 6C, c1-c5) and without any quercetin (Fig. 6C, a1-a5). In case of 1 µM quercetin supplemented flower buds, pollen tubes were found to reach the base of styles in just 2 days of pollination. Whereas in the same time period, pollen tubes grew only a way down to style but did not reach to the end of style in case of flower buds exposed to 10 nM, 20 nM and no quercetin.

### *FLS* silencing reduced endogenous free IAA content in shoot apex

**Figure 7:**
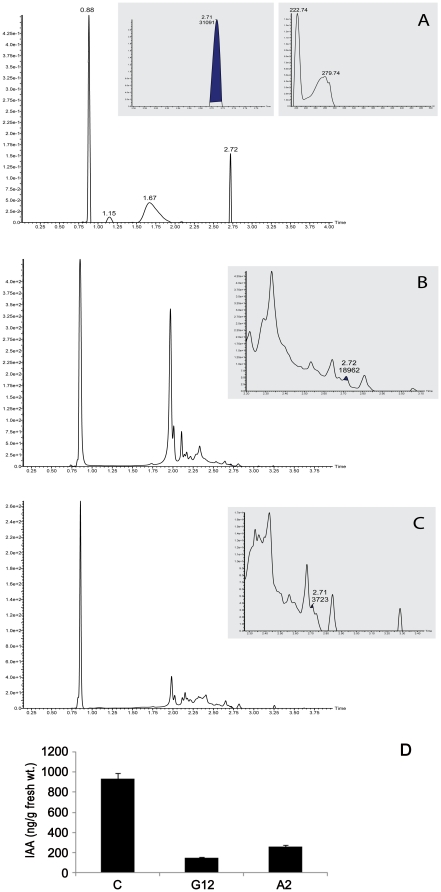
Endogenous free indole acetic acid (IAA) content in apical region of shoot of control and *FLS* silenced transgenics. Endogenous free IAA content was determined using Ultra Performance Liquid Chromatography (UPLC). A, UPLC chromatogram of 10 µg/ml IAA standard showing sharp peak at retention time (RT) of 2.72 min. Absorbance spectra of this peak was measured by photodiode array detector and was observed at 222 nm. B, Chromatogram of endogenous IAA isolated from apical portion of control tobacco shoot showing peak at RT of 2.72 min. C, Chromatogram of endogenous IAA isolated from apical portion of *FLS* silenced transgenic line (G12) showing IAA peak at RT of 2.72 min. D, Graph depicts the endogenous content of IAA measured in apical region of control (C) and silenced transgenic lines (G12 and A2). IAA was found to be reduced in silenced transgenic lines as compared to control tobacco. The quantification was performed with three replications and is represented as mean ± SD.

Flavonols has been reported as negative regulators of auxin/free IAA transport in plants [17]. Since *FLS* silencing reduced quercetin content as well as delayed flowering, its effect was analyzed on endogenous free IAA content in apical region of the plants. Endogenous IAA content of control as well as *FLS* silenced transgenic tobacco lines were determined by ultra performance liquid chromatography (UPLC). This technique has many advantages over others such as no tedious derivatization and fast analysis. UPLC chromatogram of 10 µg/ml IAA standard showed sharp peak at retention time of 2.72 min. Absorbance spectra of this peak was measured by photodiode array detector and was observed at 222 nm (Fig. 7A). Chromatogram of endogenous IAA isolated from apical portion of control tobacco shoot (Fig. 7B) and *FLS* silenced transgenics (Fig. 7C) also showed peak at retention time of 2.72 min. Endogenous IAA content was found to be significantly higher in apical region of control tobacco shoot as compared to strong *FLS* silenced transgenic lines, G12 and A2. IAA content was found to be 144 ng/g FW and 263 ng/g FW in G12 and A2 transgenic lines respectively as compared to 935 ng/g FW of control tobacco plant (Fig. 7D). Thus, *FLS* silenced lines G12 and A2 showed 85% and 72% reduction in their free IAA content respectively.

## Discussion

Post-transcriptional gene silencing (PTGS) is very efficient technology for suppressing the activity of a particular gene. Efficient gene silencing of flavonoid pathway genes have been reported earlier in *Medicago*, soybean, petunia, tobacco, cyclamen and many more plants [9], [10], [18], [19], [20]. Gene silencing machinery in plants appeared to be much more specific than in animals [21]. Therefore silencing of unintended target genes, due to small interfering RNA (siRNA) derived from *FLS* RNAi cassette, is highly unlikely but cannot be excluded. In the present study, however no putative unintended target genes were identified by *in silico* comparison of small (20–21 nucleotides) perfect matching stretches, derived from the *FLS* RNAi gene construct, to the database of tobacco submitted at NCBI.

In tobacco (*Nicotiana tabacum* cv xanthi), flavonol biosynthesis was efficiently downregulated by RNAi-mediated suppression of flavonol synthase (FLS) encoding gene. This led to a strong decrease in *FLS* gene expression in transgenic tobacco. As a consequence, more than 80% reduction in quercetin content was measured. The quercetin has been reported as a major flavonol present in tobacco [10]. The reduction in flavonol content may lead to increase in potential of flavonoid pathway towards the anthocyanins or flavan-3ols formation. Anthocyanins content was found to be decreased whereas flavan-3ols (catechin, epicatechin & epigallocatechin) content were increased in *FLS* silenced transgenic lines. Similar increase in catechin and epicatechin content has been observed earlier in *Nicotiana tabacum* by overexpression of a grape berry transcription factor VvMYB5b [22]. While an increase in anthocyanin content has been reported in petunia by altering the competition between dihydroflavonol 4-reductase and flavonol synthase [8], [9]. Interestingly, red coloured flower has also been produced in tobacco by suppression of two endogenous genes (flavanone 3-hydroxylase and flavonol synthase) and by simultaneously overexpression of gerbera DFR gene. This has resulted in increased level of pelargonidin in tobacco that produced red colour to the flowers [10]. All these studies demonstrated that FLS is a key enzyme in the regulation of flux into different branches of flavonoid biosynthesis. Importantly, no effect of *FLS* silencing is observed on the transcript expression of other genes encoding for enzymes of flavonoid biosynthetic pathway.

Flavonols, a class of flavonoids have especially been shown to have strong stimulatory effects on pollen development, germination, pollen tube growth, and seed set [2], [3], [23]. The petunia plants harbouring a complete block of flavonoids production due to antisense Chs or sense Chs cosuppression had white flowers and male sterile character [5], [16]. The inability of pollen from the sterile *wha* mutant to germinate normally has been found to be complemented by flavonol addition [5]. When flavonols applied to the stigmas, tube growth of Chs deficient sterile pollen and seed set was partly rescued. This has led to the assumption that Chs-deficient pollen lacks factors that are required for pollen tube growth. In these cases, such stigmas were functionally complemented with flavonols [24]. We observed through *in vitro* as well as *in vivo* studies a strong inhibition in pollen tube growth of self-pollinated *FLS* RNAi tobacco that has ultimately resulted in a seed set arrest. All strong *FLS* silenced transgenic tobacco lines yielded fruits with significantly less number of seeds. Earlier, *in vitro* experiments have suggested the probable effect of flavonols on pollen development, germination, and pollen tube growth. However, this is the first report documenting these specific roles of flavonol (quercetin) through both *in vitro* and *in vivo* experiments using *FLS* silenced tobacco. Silencing of *FLS* specifically blocks the flavonol synthesis in tobacco. The *in vitro* and *in vivo* experiments conducted as explained under materials and methods have found that 1 µM quercetin was sufficient to rescue the inhibited pollen germination and pollen growth in *FLS* silenced tobacco lines. These observations support the hypothesis that flavonols particularly quercetin is essential for pollen germination in tobacco. In addition, this study has also documented the application of *FLS* silencing as a novel approach to obtain less seeded fruits. In case of the tt4 (Chs) mutant of Arabidopsis (*Arabidopsis thaliana*) only a small reduction in seed set was observed and from that study flavonoids were appeared not to be essential for fertility [23], [25]. Apparently, the role of flavonoids in plant reproduction varies between different plant species.

Plant hormones play an important role in regulating fruit development and plant growth [26], [27], [28]. A possible direct role of flavonoids in auxin distribution has been proposed earlier by several research groups. Loss of CHS activity in Arabidopsis has caused an increase in polar auxin transport [13]. Additional evidence that flavonoids act as auxin transport inhibitors has been obtained from experiments with *CHS* RNAi-silenced *Medicago truncatula* plants. The flavonoid-deficient roots of these plants showed an increase in their auxin transport relative to wild type. These plants were also unable to initiate root nodulation [19]. Amongst the flavonoids, flavonols have been considered to be highly active in inhibiting auxin transport [17]. To confirm this consideration, free IAA levels were estimated in *FLS* RNAi silenced tobacco. The lower concentration of free IAA in shoot apical region of *FLS* silenced lines compared to control plants could be mainly responsible for delayed flowering and inflorescence with less number of flowers. Further, low IAA content also led to short and kinked shape of the pollen tubes of *FLS* silenced lines compared with the control tubes. The results showed that IAA is one of the most important hormones regulating pollen tube growth. Our results provide the confirmation to assumption of earlier study that IAA pays an important in the regulation of pollen germination [29].

In short, this study on *FLS* silencing in tobacco has addressed three very important points. First, flavonol quercetin is important for pollen germination and pollen tube growth in tobacco. This was evinced by the affected pollen development and pollen tube growth in *FLS* silenced tobacco that has reduced level of quercetin compared to control plant. Secondly, *FLS* silencing leads to fruits with arrested seed set or with significantly less number of seeds. This finding of the role of flavonols in seed set can be explored in two ways 1) the problem of seed set can be rescued by imparting flavonols to the plants, and 2) direct implication of this strategy could be in obtaining less-seed/seedless fruits. Third, the decrease in quercetin content has increased the polar free IAA transport towards the root. This has resulted in decreased endogenous concentration of free IAA in apical region of shoot. Thus, the delayed flowering, less flower, inhibition in pollen germination and less seeded fruits in *FLS* silenced tobacco was found to be under quercetin-mediated free IAA regulation.

## Materials and Methods

### RNA interference (RNAi) construct preparation to silence *FLS*

Two full-length cDNAs encoding flavonol synthase (*NtFLS1*; DQ435530.1 and *NtFLS*; AB289451.1) were obtained from a cDNA library of tobacco (*Nicotiana tabacum* cv Xanthi). Both sequences were aligned using CLUSTAL W program. The conserved region of 233 bp of these two sequences and a binary vector pFGC1008 was used to make an ihpRNA construct for silencing FLS in tobacco. To create an inverted repeat construct, *Asc*I restriction site at 5 and *Swa*I restriction site at 3′ end of *NtFLS* cDNA fragment was incorporated through PCR using forward primer 5′ GGCGCGCCGGTTGATCATTTGTTCCATAAG-3′ and reverse primer 5′ ATTTAAATCTTGTTGAAAATCAATTATTACC-3′ for cloning the fragment in sense orientation in between 35S promoter and GUS intron. While *Spe*I restriction site at 5′ and *Bam*HI restriction site at 3′ end of *NtFLS* cDNA fragment was incorporated through PCR using forward primer 5′ ACTAGTGGTTGATCATTTGTTCCATAAG-3′ and reverse primer 5′ GGATCCCTTGTTGAAAATCAATTATTACC-3′ to clone the fragment in antisense orientation (Fig. 1). These two fragments were first cloned individually in pGEMT-easy vector and restrict digested to produce the cohesive and blunt terminal ends. These cohesive/blunt ended fragments were then cloned in pFGC1008 vector. This has produced pFGC-FLS RNAi construct.

### Plant transformation

The resulting construct pFGC-FLS RNAi as well as an empty pFGC1008 vector containing no insert was transferred to *Agrobacterium tumefaciens* strain LBA4404 through tri-parental mating. *Agrobacterium*-mediated leaf disc co-cultivation method was used for tobacco transformation [30]. For this, tobacco seeds were firstly treated with 10% Tween-20 for 5 min and with 70% ethanol for 30 sec followed by surface sterilization with 0.001% mercuric chloride for 3 min and washed thrice with sterile distilled water. Tobacco plants were allowed to grow from seeds for about one month and were transformed via *A. tumefaciens* LBA4404 strain containing pFGC-FLS RNAi construct. On the other hand, the control plants were generated by transforming tobacco leaf discs via *A. tumefaciens* strain LBA4404 containing an empty pFGC1008 vector. The transformed plants were selected on MS medium containing 50 mg/l of hygromycin as plant selection antibiotic at 25°C [31]. Several transgenic tobacco lines were screened for transgene integration via PCR using vector specific primers. These transgenics were allowed to grow in greenhouse to set seeds by self-pollination. The seeds of transgenic T1 plant lines were selected on MS media containing 50 mg/l of hygromycin. Resistant plants were transferred to a closed greenhouse to produce flowers and for use in further analyses.

### Expression analysis of flavonoid biosynthetic pathway genes

Semi-quantitative RT-PCR analysis was performed to test the effect of *FLS* silencing on the endogenous expression levels of flavonoid biosynthetic pathway genes. For this, total RNA was extracted from the leaf of control and silenced transgenics using RNeasy plant mini kit (Qiagen). First strand cDNA was synthesized from 1 µg of total RNA using SuperScript III reverse transcriptase (Invitrogen) according to the supplier's instructions. The cDNA was treated with RNase H to remove left over RNA template. This cDNA was used as a template for relative PCR where a set of selected primers of flavonoid biosynthetic pathway genes as well as internal control were used. The list of selected flavonoid biosynthetic pathway genes, their accession number and oligonucleotide primers used in the study are provided as Table S1. Linearity between the amount of input RNA and the final RT-PCR products was verified and confirmed. After standardizing the optimal amplification at exponential phase, PCR was carried out under the conditions of 94°C-4 min, 94°C-30 s, 50 to 60°C-40 s (57°C for CHI, F3H & FLS, 54°C for CHS and 53°C for ANS) for 25 cycles. For each transgenic line, expression of all flavonoid pathway genes was measured in triplicate. The 26S rRNA based gene primers were used as internal control for expression studies [32].

### In vitro pollen germination and pollen rescue assays

For *in vitro* pollen germination assay, pollen grains from freshly dehiscent anthers of the control as well as the *FLS* silenced transgenic lines were collected. These pollens were dabbed on slides that were covered with solid pollen germination medium (GM) containing 1 mM CaCl_2_, 1 mM H_3_BO_4,_ 1 mM MgSO_4_.7H_2_O, 1 mM KNO_3,_ 0.25 mM sucrose and 1% agar as described previously [33]. Thereafter, slides were incubated at 28°C with 100% moisture. Pollen germination ratio was determined by light microscope attached with Nikon digital camera (Dxm 1200). To determine germination frequency, usually 500 pollen grains were counted. Tube length of 50 to 100 pollen grains was measured. Only tube longer than half the size of a pollen grain was judged as germinated. Each experiment was repeated at least three times.

Flavonol induced *in vitro* pollen germination rescue assay was performed as described earlier [34]. For rescue assays, pollens from flowers of *FLS* silenced transgenic lines were germinated on GM only (as control) and on GM supplemented with 10 nM, 20 nM & 1 µM quercetin separately. Pollen germination frequency was determined as described above. The flavonol quercetin used in this experiment was purchased from Sigma. It was dissolved in DMSO immediately before addition to the media. In control experiments, only DMSO was dissolved to the media. Importantly, no effect of DMSO (<0.3%) was observed on pollen germination.

### In vivo pollen germination and pollen rescue assays

For *in vivo* pollen germination assays, mature closed flowers of control and *FLS* silenced transgenics were emasculated and pollinated. Two days after pollination, pistils were harvested and incubated overnight at 60°C in 1 M KOH. After rinsing with water, pistils were transferred to a microscope slide and stained with 0.005% aniline in 50% glycerol according to the described procedure [6]. A cover slip was placed on top and pressed gently. Callose in the pollen tubes was visualized by UV-1A filter on a Nikon eclipse 80i florescent microscope and photographed using Nikon digital camera Dxm 1200 C.

For *in vivo* pollen rescue assays, flower buds at stage II (the stage representing the middle of bud formation and full flower opening as shown in the Fig. 6B) of *FLS* silenced transgenic lines were exposed to pollen maturation media only and to media containing different concentrations of quercetin i.e. 10 nM, 20 nM & 1 µM quercetin. Flowers after maturation were emasculated and pollinated. Pistils of flowers exposed to various quercetin treatments were stained for callose visualization as mentioned above.

### Flavonoid analysis by HPLC

Catechin, epi-catechin and epi-gallocatechin contents were analyzed in leaf tissues of control and *FLS* silenced transgenic tobacco following the earlier described method [35]. Water's HPLC with a C-18 reverse phase 250×4.0 mm, 5 µm column was used for these analyses. Leaf samples were crushed and dried up to a constant weight. Dried sample (0.1 g) was extracted thrice with 2.5 ml of 70% aqueous methanol followed by 1.5 ml and 1 ml of 70% aqueous methanol. This extract was centrifuged with 4000 rpm at 4°C for 10 min. Supernatant was collected and volume was made up to 5 ml by 70% methanol. The extract was passed through Millipore filter (0.45 µm) prior to injection in HPLC. The mobile phase finally adopted for catechin, epicatechin and epi-gallocatechin was acetonitrile/0.1% ortho-phosphoric acid in water (w/v) with a flow rate of 1 ml/min and these contents were detected at 280 nm.

The flavonol (quercetin) estimation was performed according to the method described earlier [35]. Hundred milligrams of dried leaves from control and *FLS* silenced transgenic tobacco were firstly extracted with 2 ml of 70% methanol. After centrifugation at 6000 rpm for 10 min at 4°C, supernatant was collected and the residue was extracted twice with 1.5 ml of 70% methanol. The filtrate was evaporated to 1.0 ml, and 3 volumes of HCl (1 M) were added followed by incubation at 94°C for 2 h to hydrolyze any conjugate forms of flavonoids. After hydrolyzation, samples were extracted with ethyl acetate, evaporated to dryness, and resuspended in 80% methanol. The sample was filtered through a 0.45* µ*m filter prior to injection into HPLC. The mobile phase used for quercetin estimation was consisted of 0.05% trifluoroacetic acid (TFA) in water/acetonitrile and the detection wavelength was set at 355 nm.

### Photometric determination of anthocyanins

Extraction of anthocyanins from flowers of control and *FLS* silenced transgenic tobacco lines was performed following the protocols of [36], [37] with minor modifications. One milliliter of acidic methanol (1% HCl, v/v) was added to 300 mg of fresh plant material. Samples were incubated for 18 h at room temperature under moderate shaking. Plant material was sedimented by centrifugation (14,000 rpm, room temperature, 1 min) and 400 µl of the supernatant was added to 600 µl of acidic methanol. Absorption of the extracts was determined using UV-Vis spectrophotometer at 530 nm and 657 nm. Quantification of anthocyanins was performed using the following equation: Q_Anthocyanins_  =  (A_530_ − 0.25× A_657_)×M^−1^, where Q_Anthocyanins_ is the amount of anthocyanins, A_530_ and A_657_ are the absorptions at the indicated wavelengths and M^−1^ is the weight (g) of the plant material used for extraction. All samples were measured as triplicates in two independent biological replicates. Error bars indicate SD of the average of anthocyanin content.

### Free IAA estimation by UPLC

The apical shoot region of control and *FLS* silenced transgenic tobacco lines was used for free IAA estimation by liquid chromatography following the described method [38]. Five gram of tissue was chopped and homogenized at high speed for 3 min with 30 ml of 80% cold methanol. The filtrate was evaporated to 10 ml at below 40°C. The residue was dissolved in 20 ml of acetic ether (pH 2.8, adjusted with 0.1 mol/l HCl) and transferred to a separating funnel. After shaking intensely for 1 min, the organic phase was collected. To this, 20 ml of buffer solution (pH 7) was added and oscillated. The buffer was prepared by mixing 219 ml of 0.1 mol/l NaOH with 250 ml of 0.2 mol/l NaH_2_PO_4_ and diluting the mixed solution to 1000 ml with water. The aqueous phase was again extracted with 20 ml of acetic ether (pH 2.8). Finally, the upper layer was evaporated to near dryness at below 40°C. The residue was redissolved in 2 ml of acetonitrile and filtered through a 0.22 µm filter before UPLC analysis.

The Water's Aquity Ultra Performance Liquid Chromatography system consisted of quaternary pump, a diode-array detector and MassLynxV4.1 system was used for free IAA analysis. Chromatographic separation was performed on BEH C18 column (1.7 µm; 2.1×100 mm) at 22°C. The acetonitrile and water (45∶55, v/v %) was used as mobile phase. The elution was performed at a flow rate of 0.5 ml/min. The injection volume of sample was 5 µl for each analysis. The UV detection wavelength was set at 222 nm. MassLynxV4.1 software was used to control the whole LC system.

## Supporting Information

Figure S1 Nucleotide sequence alignment of flavonol synthase cDNA, NtFLS1 (DQ435530.1) and NtFLS (AB289451.1) from *Nicotiana tabacum*.
(TIF)

Figure S2 Overall pictorial view of gel picture showing FLS fragment cloned in pFGC1008 in sense and antisense orientation.
(TIF)

Figure S3 Genomic DNA PCR confirmation of inserted FLS RNAi cassette in tobacco transgenic lines.
(TIF)

Figure S4 Expression analysis of flavonoid biosynthetic pathway genes in seedlings (shoot and root) of control and *FLS* silenced transgenic line (G12) as determined by reverse transcriptase PCR (RT-PCR).
(TIF)

Table S1 Primers for RT-PCR analysis of flavonoid biosynthetic pathway genes.
(DOC)
