# Supplementary figures and images for: Post-Transcriptional Silencing of Flavonol Synthase mRNA in Tobacco Leads to Fruits with Arrested Seed Set
Source: PLoS One. 2011 Dec 1;6(12):e28315. doi: 10.1371/journal.pone.0028315 (PMC3228754; doi:10.1371/journal.pone.0028315)

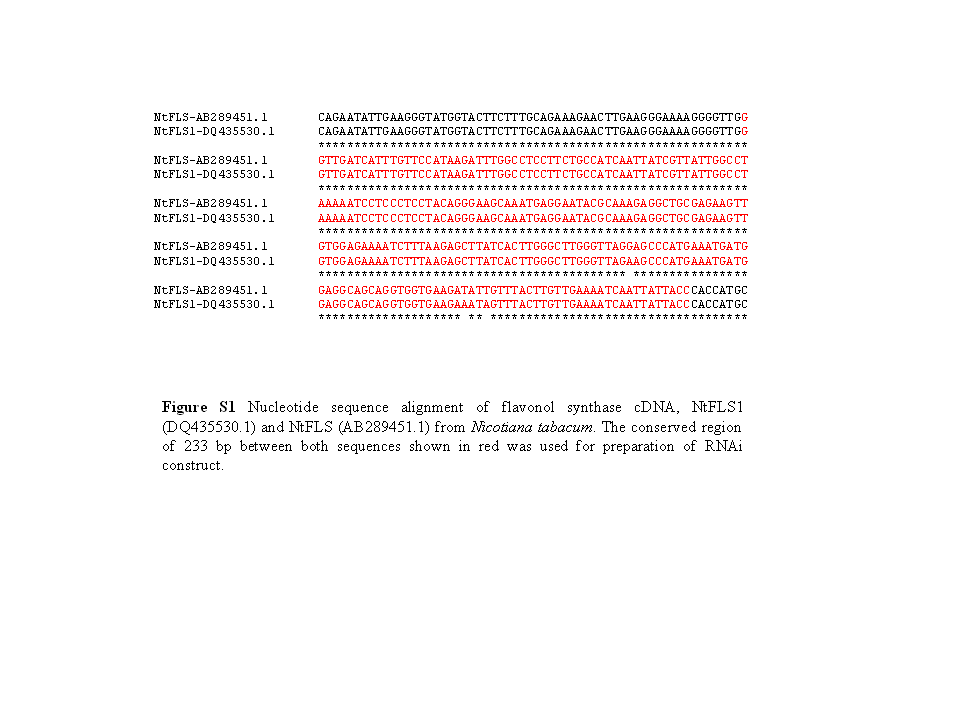

Supplement: Figure S1 — Nucleotide sequence alignment of flavonol synthase cDNA, NtFLS1 (DQ435530.1) and NtFLS (AB289451.1) from Nicotiana tabacum. (TIF) [file pone.0028315.s001.tif]

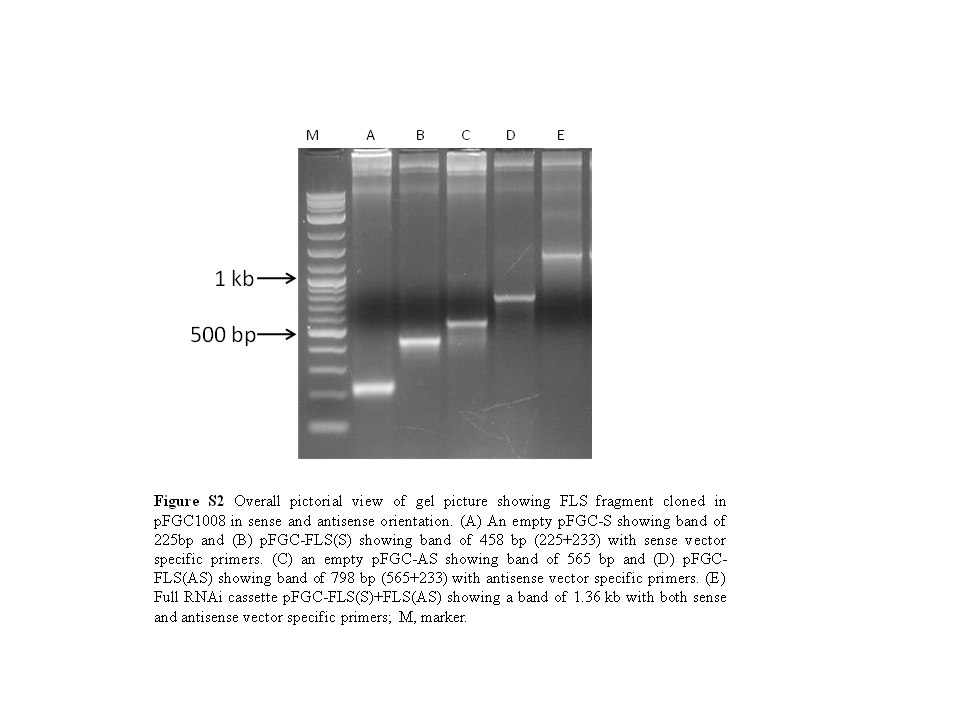

Supplement: Figure S2 — Overall pictorial view of gel picture showing FLS fragment cloned in pFGC1008 in sense and antisense orientation. (TIF) [file pone.0028315.s002.tif]

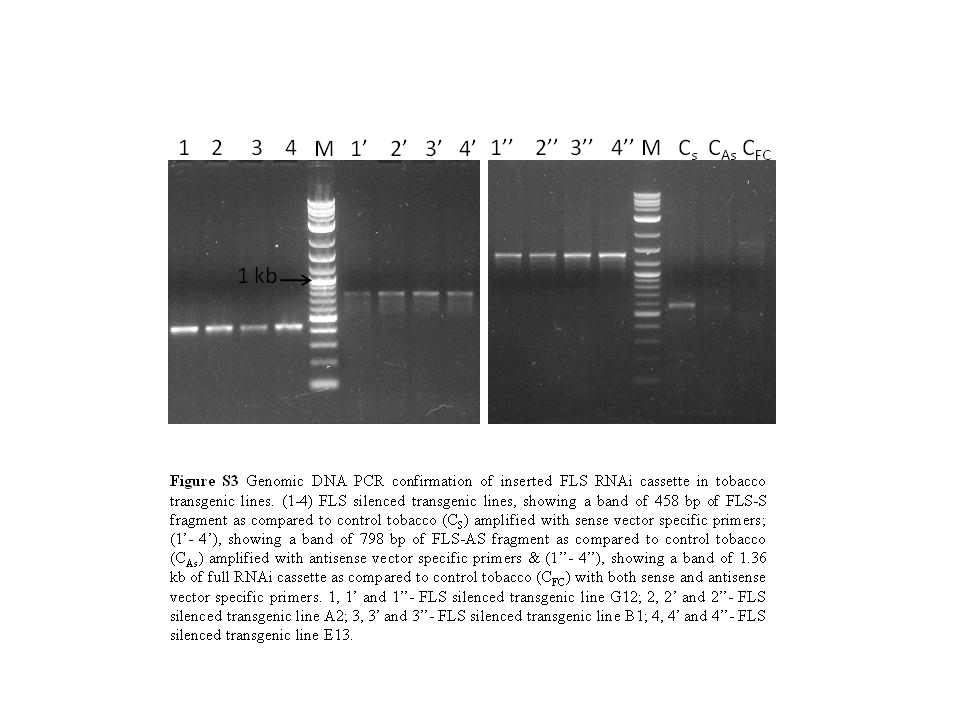

Supplement: Figure S3 — Genomic DNA PCR confirmation of inserted FLS RNAi cassette in tobacco transgenic lines. (TIF) [file pone.0028315.s003.tif]

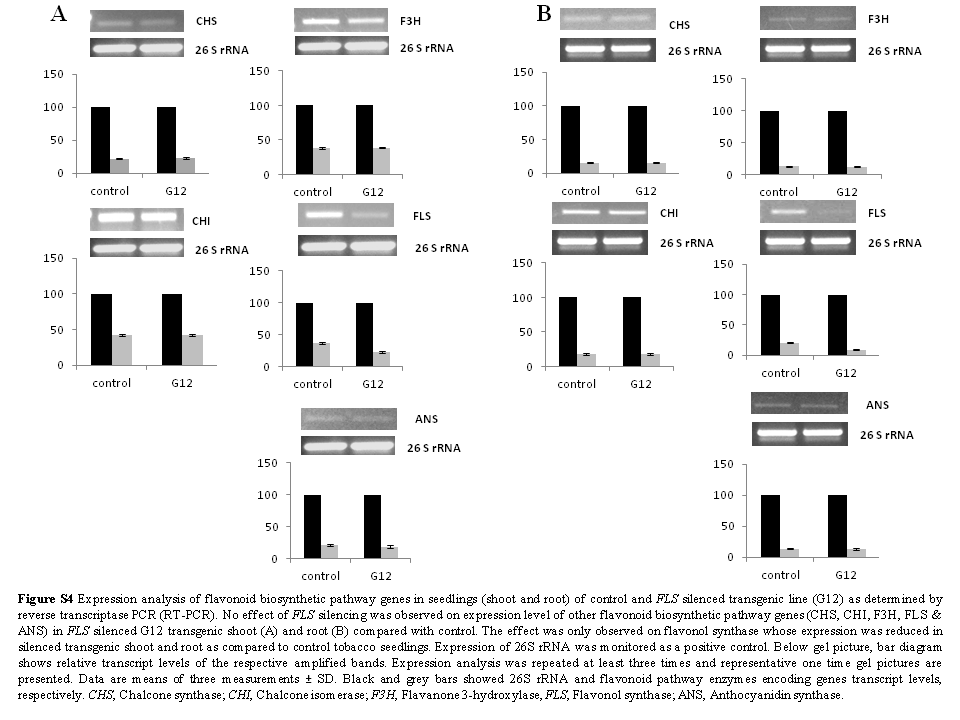

Supplement: Figure S4 — Expression analysis of flavonoid biosynthetic pathway genes in seedlings (shoot and root) of control and FLS silenced transgenic line (G12) as determined by reverse transcriptase PCR (RT-PCR). (TIF) [file pone.0028315.s004.tif]
