# Supplementary material for: Post-Transcriptional Silencing of Flavonol Synthase mRNA in Tobacco Leads to Fruits with Arrested Seed Set
Source: PLoS One. 2011 Dec 1;6(12):e28315. doi: 10.1371/journal.pone.0028315 (PMC3228754; doi:10.1371/journal.pone.0028315)
Supplement: Table S1 — Primers for RT-PCR analysis of flavonoid biosynthetic pathway genes. (DOC) [file pone.0028315.s005.doc]

**Table S1** Primers for RT-PCR analysis of flavonoid biosynthetic pathway genes

| **Genes** | **Accession No.** | **Forward primer (5**ʹ**-3**ʹ**)** | **Reverse primer (5**ʹ**-3**ʹ**)** |
| --- | --- | --- | --- |
| Nt*CHS* | AB311783.1 | GTACAACTAGTGGTGTAGACA | CCAACTTCACGAAGGTGAC |
| Nt*CHI* | AB213651.1 | CGAGTGACTATGATCTTGCC | CTGACGCGTCGGCATAGC |
| Nt*F3H* | AB289450.1 | GGTAGTTGATCATGGTGTTGA | GTTCCTGGATCAGTGTCTCG |
| Nt*FLS* | DQ435530.1 and AB289451.1 | GTCCACAACGTTGCATGGTG | CACAACTTCTCGCAGCCTC |
| Nt*ANS* | AB289447 | CGAGGACAAGTGCGACTTAT | GAGATTCTTACTTTCTCTTTATT |
| 26S rRNA | Conserved region of 26S rRNA encoding genes | CACAATGATAGGAAGAGCCGAC | CAAGGGAACGGGCTTGGCAGAATC |
